# Intrinsic predisposition of naïve cystic fibrosis T cells to differentiate towards a Th17 phenotype

**DOI:** 10.1186/1465-9921-14-138

**Published:** 2013-12-17

**Authors:** Rahul Kushwah, Stéphane Gagnon, Neil B Sweezey

**Affiliations:** 1 McMaster Stem Cell and Cancer Research Institute (SCC-RI), Faculty of Health Sciences, McMaster University, 1280 Main Street West, Hamilton, Ontario, Canada L8S 4K1; 2 Physiology and Experimental Medicine, Research Institute, The Hospital for Sick Children, 555 University Avenue, Toronto, Ontario, Canada M5G 1X8; 3 Departments of Paediatrics and Physiology, and the Institute of Medical Sciences, University of Toronto, 555 University Avenue, Toronto, Ontario, Canada M5G 1X8

**Keywords:** Cystic fibrosis, Naïve T cells, Th17 phenotype

## Abstract

**Background:**

Cystic fibrosis (CF) is a complex, multi-system, life-shortening, autosomal recessive disease most common among Caucasians. Pulmonary pathology, the major cause of morbidity and mortality in CF, is characterized by dysregulation of cytokines and a vicious cycle of infection and inflammation. This cycle causes a progressive decline in lung function, eventually resulting in respiratory failure and death. The Th17 immune response plays an active role in the pathogenesis of CF pulmonary pathology, but it is not known whether the pathophysiology of CF disease contributes to a heightened Th17 response or whether CF naïve CD4+ T lymphocytes (Th0 cells) intrinsically have a heightened predisposition to Th17 differentiation.

**Methods:**

To address this question, Th0 cells were isolated from the peripheral blood of CF mice, human CF subjects and corresponding controls. Murine Th0 cells were isolated from single spleen cell suspensions using fluorescence-activated cell sorting. Lymphocytes from human buffy coats were isolated by gradient centrifugation and Th0 cells were further isolated using a human naïve T cell isolation kit. Th0 cells were then assessed for their capacity to differentiate along Th17, Th1 or Treg lineages in response to corresponding cytokine stimulation. The T cell responses of human peripheral blood cells were also assessed *ex vivo* using flow cytometry.

**Results:**

Here we identify in both mouse and human CF an intrinsically enhanced predisposition of Th0 cells to differentiate towards a Th17 phenotype, while having a normal propensity for differentiation into Th1 and Treg lineages. Furthermore, we identify an active Th17 response in the peripheral blood of human CF subjects.

**Conclusions:**

We propose that these novel observations offer an explanation, at least in part, for the known increased Th17-associated inflammation of CF and the early signs of inflammation in CF lungs before any evidence of infection. Moreover, these findings point towards direct modulation of T cell responses as a novel potential therapeutic strategy for combating excessive inflammation in CF.

## Background

Cystic Fibrosis (CF) is an autosomal recessive disorder caused by mutations within the CF transmembrane conductance regulator (*CFTR*) gene [1,2]. Initially identified in the apical membranes of epithelial cells, defects in CFTR expression associated with chloride channel defects [3] have also been found in circulating T lymphocytes [4]. Pulmonary disease, the major cause of morbidity and mortality in CF [5], is characterized by dysregulation of cytokines and a vicious cycle of infection and inflammation which causes a progressive decline in lung function, eventually resulting in respiratory failure and death (reviewed by [6,7]). The lung disease can be particularly exacerbated by *P. aeruginosa* infections [8]. Th17 is a recently identified helper T cell subset identified by production of interleukin (IL)-17 [9]; it has been linked to the pulmonary exacerbations and neutrophilia observed in CF [10,11], including neutrophilia very early in life [12]. CF patients with active *P. aeruginosa* infections have elevated levels of Th17 cytokines in their sputum and studies have identified the Th17 cytokine IL-23 as a major factor in orchestrating *P. aeruginosa* - induced pulmonary inflammation [10]. The pulmonary Th17 response, particularly IL-17 levels, predicts future acquisition of *P. aeruginosa* infections [13]. In a murine model of CF, the Th17 response has also been described as detrimental to clearance of *A. fumigatus*, a fungus which often exacerbates CF lung disease through the associated condition allergic bronchopulmonary aspergillosis [14]. Although studies have identified an active role of Th17 response in modulating CF pulmonary pathology, the underlying mechanism(s) specifically promoting a Th17 response are not understood. It is not known whether the pathophysiology of CF disease contributes to a heightened Th17 response, or whether naïve CF T cells are intrinsically prone to Th17 differentiation.

In this study, we identified an innate predisposition of naïve CF T cells in both humans and mice to selectively undergo differentiation into the Th17 lineage while retaining a normal predisposition to other helper T cell lineages. These findings identify an intrinsic defect in CF T cells, which independently of the cytokine milieu in the CF lung may initiate, contribute to, or perhaps even substantially account for, the detrimental Th17 response observed in CF patients.

## Methods

### Subject characteristics

Five female CF subjects were diagnosed by repeated sweat testing using the method of Gibson and Cooke [15]. Each had two recognized disease - causing *CFTR* mutations: two were F508del homozygotes and the other three were compound heterozygotes, F508del/2183AA->G, F508del/2622+1G->A and G542X/R560T. All of these mutations are classified as severe mutations, producing very little or no functional CFTR. They were not receiving any systemic corticosteroids, were clinically stable, free of acute pulmonary exacerbation and free of signs of viral illness, and aged 15 to 22 years at the time of blood sampling. One was chronically infected with *Pseudomonas aeruginosa,* but the other four were not. Rather, their sputum cultures were positive for *Staphylococcus aureus*. The three healthy controls were two females aged 22 and 35, and one male aged 57 years. Human subject experiments were conducted according to the principles expressed in the Helsinki Declaration. All subjects (CF and controls) gave informed consent and the study was approved by the Research Ethics Board of The Hospital for Sick Children, Toronto, ON.

### CF mice

Inbred congenic mice homozygous for the F508del CFTR mutation (*CFTR-/-*, CF knock-in mice) on a C57BL/6 background and littermate controls (*CFTR+/+*, wildtype) were obtained from Dr. Christine Bear at The Hospital for Sick Children Research Institute. The mice were handled according to the Guidelines of the Canadian Council on Animal Care in science, and the protocols of the mouse experiments were approved by the Animal Care Committee, Research Institute, The Hospital for Sick Children.

### Isolation of T cells

Mouse naïve CD4+ T cells, defined as CD3+CD4+CD25- cells [16], were isolated by fluorescence-activated cell sorting (FACS Aria II, BD Biosciences, Mississauga, ON) from single spleen cell suspensions from *CFTR+/+* and *CFTR-/-* mice. Lymphocytes from human buffy coats were isolated by gradient centrifugation in Lymphoprep (Axis-Shield, Oslo, Norway) following the manufacturer’s instructions. Human naïve T cells, defined as CD3+CD4+CD25-CD45RA+CD45RO- [17], were isolated using a human naïve T cell isolation kit (Miltenyi Biotec, Auburn, CA) following manufacturer’s instructions, with purity in excess of 95%. The isolation of naïve human T cells was performed in a two step process. The first step was a negative selection of non-CD4+ T cells along with CD45RO+ T cells, which negatively selected for both memory and effector T cells, and the second step was a positive selection for CD45RA+ T cells for isolation of naïve T cells.

### Assessment of peripheral blood T cell response *ex vivo*

Mononuclear cells from human blood were cultured on plates (BD Biosciences) coated with CD3 antibody (clone HIT3a) in the presence of CD28 antibody (clone CD28.2, eBiosciences, San Diego, CA) for 4 days. On day 4, cells were treated with 50 ng/mL PMA and 1 μM Ionomycin (Sigma-Aldrich, Oakville, ON) for 6-8 hr followed by analysis for IL-17 and IFN-γ production by CD4+ T cells using flow cytometry [18].

### *In vitro* differentiation of T cells

Naïve CD4+ T cells from *CFTR+/+* and *CFTR-/-* mice were differentiated *in vitro* into IFN-γ- producing Th1 cells [19], into Foxp3+ regulatory T (Treg) cells [20] or into the IL-17- producing Th17 lineage as described previously [21]. Production of IFN-γ and IL-17 by differentiated mouse T cells was confirmed using respective ELISA kits following manufacturer’s instructions (R&D Systems, Minneapolis, MN). *In vitro* naïve human T cell differentiation was carried out by culturing cells in a plate coated with anti-CD3 antibody (5 μg/mL) for 6-7 days with anti-CD28 (2 μg/mL) in the presence of IL-6 (50 ng/mL), IL-23 (25 ng/mL), IL-1β (10 ng/mL), TGF-β1 (1 ng/mL; Peprotech, Rocky Hill, NJ), anti-IL-4 (clone MP4-25D2; 10 mg/mL) and anti-IFN-γ (10 mg/mL, clone NIB42; eBiosciences) for Th17 differentiation, or TGF-β1 (5 ng/mL; Peprotech) for Treg differentiation.

### Statistical analysis

Student two-tailed *t* test was used for statistical analysis. A *p* value <0.05 was considered significant.

## Results and discussion

### Naïve CFTR-/- CD4+ T cells preferentially undergo Th17 differentiation

**Figure 1:**
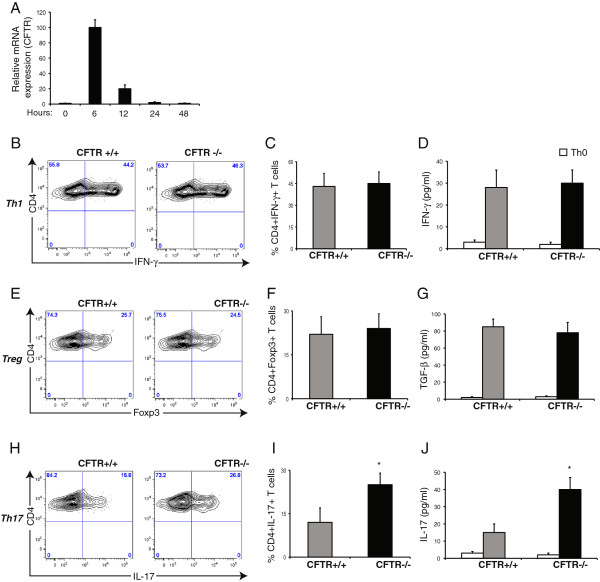
**CFTR-/- T cells are intrinsically predisposed to differentiate along the Th17 lineage. (A)** Relative mRNA expression of CFTR in naїve CD4+ T cells from *CFTR+/+* mice following stimulation with anti-CD3 and anti-CD28 antibodies at different time points. **(B-J)** Naїve CD4+ T cells from *CFTR+/+* and *CFTR-/-* mice were differentiated into Th1, Treg and Th17 lineages respectively in the presence of inducing cytokines. **(B)** Flow cytometry plots of IFN-γ production by CD4+ T cells. **(C)** Proportions of IFN-γ producing cells generated under Th1 inducing conditions. **(D)** Levels of IFN-γ released by differentiated cells. **(E)** Flow cytometry plots of Foxp3 expression by CD4+ T cells. **(F)** Proportions of Foxp3+ cells generated under Treg inducing conditions. **(G)** Levels of TGF-β released by differentiated cells. **(H)** Flow cytometry plots of IL-17 production by differentiated cells. **(I)** Proportions of IL-17 producing cells generated under Th17 inducing conditions. **(J)** Levels of IL-17 released by differentiated cells. n = 4-6 mice per group, representative of 2-3 independent experiments. Mean ± SEM, * p < 0.05 vs *CFTR +/+.*

Initially, CFTR expression was thought to be restricted to epithelial cells [22,23], but later studies confirmed that CFTR is also expressed in lymphocytes [4]. Moreover, T cell clones derived from CF subjects show a defective c-AMP regulated chloride current, pointing towards a functional role of CFTR in regulating T cell function [4]. Differentiation of naïve CD4+ T cells into different effector lineages, such as Th17 cells, is directed primarily by the local cytokine environment in the presence of T cell receptor activation and co-activation of co-stimulatory molecules [24,25]. We assessed the kinetics of CFTR expression during naïve CD4+ T cell differentiation. Naïve CD4+ T cells were isolated from *CFTR+/+* mice and stimulated *in vitro* with antibodies to drive T cell receptor activation. On quantitative polymerase chain reaction analysis, CFTR expression is induced up to 100 fold following 6 hours of stimulation, subsequently returning to basal levels within 48 hr (Figure 1A). By contrast, we failed to observe inducibility of CFTR mRNA expression in T cells isolated from *CFTR-/-* mice (data not shown). The inducibility of CFTR expression in naïve T cells following antibody stimulation led us to hypothesize that defective CFTR may affect T cell differentiation into different lineages. Naïve CD4+ T cells from *CFTR+/+* and *CFTR-/-* mice were differentiated into IFN-γ- producing Th1 cells [19]*in vitro* (Figure 1B-D). Similar proportions of naïve CD4+ T cells underwent Th1 differentiation, which was further confirmed by using ELISA to measure levels of IFN-γ released into the media (Figure 1B-D). Similar results were identified following differentiation of naїve CD4+ T cells into Foxp3+ regulatory T cells [20], whereby both *CFTR+/+* and *CFTR-/-* naïve CD4+ T cells displayed comparable propensities to undergo Treg differentiation, by analysis of Foxp3 expression (Figure 1E, F) as well as levels of TGF-β production (Figure 1G). However, when cells were differentiated along the IL-17-producing Th17 lineage [21], 16% of naïve T cells from *CFTR+/+* mice differentiated into IL-17 producing cells, whereas Th17 differentiation of naïve *CFTR -/-* T cells was almost 2 fold higher with approximately 25-30% cells undergoing differentiation into IL-17-producing cells (Figure 1H, I). This was further confirmed by measuring IL-17 production using ELISA, which identified that *in vitro* differentiated *CFTR-/-* T cells produce significantly higher amounts of IL-17 than *CFTR+/+* T cells differentiated along a Th17 lineage (Figure 1J). Therefore, data from mice identified an intrinsic predisposition of naïve *CFTR-/-* T cells to preferentially undergo Th17 differentiation.

### Active Th17 response in the peripheral blood of CF subjects

**Figure 2:**
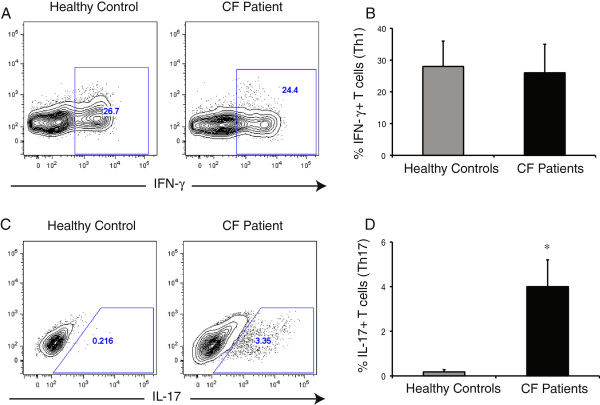
**CF subjects have an active Th17 response, which can be detected in peripheral blood. (A)** Representative flow cytometry plots of IFN-γ production by peripheral blood T cells from healthy controls and CF subjects. **(B)** Proportions of IFN-γ producing Th1 cells in peripheral blood. **(C)** Representative flow cytometry plots of IL-17 production by peripheral blood T cells from healthy controls and CF subjects. **(D)** Proportions of IL-17- producing Th17 cells in peripheral blood. 5 CF subjects and 3 healthy controls, representative of 3-4 independent experiments. Mean ± SEM. *p < 0.05.

Although studies have identified an active Th17 profile in the lungs of CF patients [13,26], an active Th17 response has not been demonstrated in the peripheral blood. Based on our findings in the above CF mouse studies suggesting a predisposition in *CFTR-/-* T cells, we postulated that an intrinsic predisposition to undergo Th17 differentiation should lead to the presence of an identifiable Th17 response in the peripheral blood of CF subjects. Peripheral blood mononuclear cells were isolated from CF subjects and healthy individuals and subsequently stimulated *in vitro*. Analysis of the Th1 response, as assessed by IFN-γ production, revealed similar proportions of IFN-γ- producing T cells in the peripheral blood of healthy individuals as well as CF subjects (Figure 2A, B). However, healthy individuals showed an extremely low frequency of IL-17- producing T cells (0.2%), which was almost 17 fold higher in CF subjects with approximately 3-4% of T cells showing IL-17 production (Figure 2C, D). These findings clearly identified an active Th17 response in the peripheral blood of CF subjects. An active Th17 response in the lung has been identified as predictive of *P. aeruginosa* infection, which promotes exacerbation of pulmonary disease [13]. Although such a predictive biomarker would be useful, it is limited by the requirement for bronchoalveolar lavage (BAL) [13]. It remains to be determined if prediction of lung infection with *P. aeruginosa* infection can be achieved using the Th17 profile in peripheral blood, bypassing the need for BAL.

### Naïve CD4+ T cells in human CF preferentially undergo Th17 differentiation

**Figure 3:**
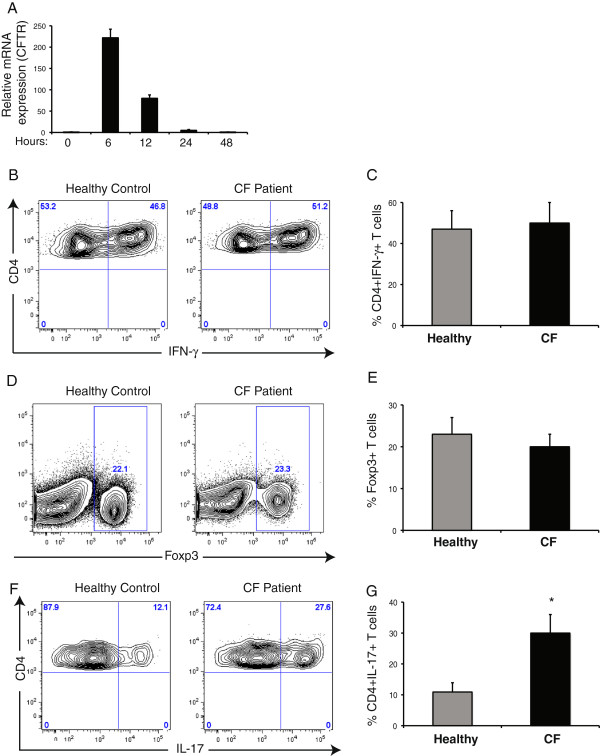
**Naїve T cells from CF subjects are intrinsically predisposed to Th17 differentiation. (A)** Relative mRNA expression levels of CFTR on naїve human CD4+ T cells following antibody stimulation at different time-points. **(B)** Representative flow cytometry plots of IFN-γ production by naїve T cells from healthy controls and CF subjects following Th1 differentiation. **(C)** Proportions of IFN-γ- producing Th1 cells. **(D)** Representative flow cytometry plots of Foxp3 expression by naїve T cells from healthy controls and CF subjects following differentiation into Tregs. **(E)** Proportions of Foxp3+ Tregs. **(F)** Representative flow cytometry plots of IL-17 production by naїve T cells from healthy controls and CF subjects following Th17 differentiation. **(G)** Proportions of IL-17- producing Th17 cells. 5 CF subjects and 3 healthy controls, representative of 3-4 independent experiments. Mean ± SEM. *p < 0.05.

There have been clear differences documented between murine and human CF in terms of pulmonary inflammation, and thus observations from murine CF may not always translate to human CF. Therefore, we wanted to confirm inducibility of human CFTR in naïve human T cells following antibody stimulation, as was observed in murine CF. CFTR was induced approximately 200 fold at the mRNA level following 6 hr of stimulation of naïve human CD4+ T cells and normalized to basal levels within 48 hr (Figure 3A), consistent with our mouse CF observations (Figure 1A). Next we wanted to identify whether, similar to mouse CF, T cells from human CF subjects are intrinsically predisposed towards Th17 differentiation whilst having a normal propensity for Th1 and Treg differentiation. Under conditions known to selectively drive Th1 differentiation, approximately 50% of naïve T cells both from CF subjects and from healthy individuals differentiated into IFN-γ- producing Th1 cells (Figure 3B, C). Similarly, in the presence of TGF-β [27], T cells from both healthy individuals and CF patients showed a similar propensity to Treg generation with almost 20% of the cells differentiating into Foxp3+ Tregs [27,28] (Figure 3D, E). Although only 12% of naïve T cells from healthy individuals differentiated into IL-17- producing Th17 cells, this proportion was roughly doubled in CF subjects, with approximately 27% of naïve CD4+ T cells undergoing differentiation into Th17 cells (Figure 3F, G). These findings confirm our observations from CF mice and clearly indicate the preferential predisposition of naïve T cells in CF to undergo differentiation along a Th17 lineage.

Contamination of naïve CD4+ T cells with memory T cells could affect our results. Our strategy for isolation was based on depletion of CD45RO+ cells. CD45RO is not expressed exclusively on memory T cells, but is also expressed on effector T cells [29]. Therefore, depletion of CD45RO+ cells leads to depletion of both the memory as well as effector T cell subsets. Lack of CD45RO+ cells was employed to confirm the lack of memory T cells in isolated naïve CD4+ T cells. Moreover, the proportions of IL-17- producing T cells in the peripheral blood were in the range of 0.2-3%, whereas following differentiation of naïve T cells into IL-17- producing T cells, the proportions ranged between 10-30%. Polyclonal T cell activation was used for differentiation of naïve T cells into IL-17- producing T cells as well as for activation of peripheral T cells. If contamination with memory T cells had been affecting the data in our experiments, we would then expect the proportions of IL-17- producing T cells should have been comparable between peripheral blood and following naïve T cell differentiation. However, the 10-fold higher proportions of IL-17- producing T cells following naïve T cell differentiation indicates that these were likely the naïve T cells which underwent differentiation into Th17 cells and not just memory T cells, because if memory T cells underwent proliferation then the proportions of IL-17-producing cells should have been comparable between the two experiments.

Previous studies have identified an active Th17 response in the CF lung, thought likely to be orchestrated by the local pulmonary environment [12-14]. In accord with these findings, we report a Th17 response in the peripheral blood of CF subjects, providing a potential biomarker of the severity of CF disease through measurement of Th17 in the peripheral blood.

Dysregulation of cytokines has been reported in CF airway epithelial cells, recently reviewed by Cohen-Cymberknoh *et al.*, [30] and, in distinct patterns, in the neutrophils of the CF lung and circulating blood [31]. To the best of our knowledge, none of the studies conducted to date have looked at the differentiation ability of naïve human CF CD4+ T cells to helper T cell lineages compared to cells from healthy subjects. At a time before the Th17 subset of T helper cells was recognized, Moss *et al.*[32] reported activated, freshly isolated, peripheral CD4+ T lymphocytes had decreased IFN-γ secretion in CF compared to control cells, prompting them to suggest a link between CF genotype and T cell cytokine dysregulation. However, it is important to note that the T cells isolated in that study were CD4+ T cells which had already differentiated within the subjects into cytokine-producing cells [32]. Hence, differences in cytokine production by those cells compared to cells from healthy controls likely reflected the differences in the ongoing T cell response in CF subjects and did not directly implicate a role of CF genotype in mediating T cell function. In our present study, naïve CD4+ T cells from CF subjects differentiated *in vitro* into IFN-γ- producing Th1 cells to an extent similar to naïve CD4+ T cells from healthy controls, further indicating that the findings reported by Moss *et al.* were not indicative of a role of CF genotype in regulating overall T cell function.

The human controls in our present study were older than the CF subjects (22, 35 and 57 years compared to 15-22 years). Li *et al.*[33] identified a decline in miR-181a microRNA as being responsible for reduced CD4+ T cell function observed with aging. However, the difference was seen in T cells from individuals who were over 70 years old and in our study, none of the CF subjects or the controls met this criterion. Moreover, the levels of IFN-γ-producing Th1 cells as well as Foxp3+ regulatory T cells were similar amongst both CF subjects as well as controls in our study and it was only the Th17 response which was selectively exaggerated in CF. Our murine data also indicated that only the Th17 response was affected in CF. In the unlikely scenario that our observations were age dependent, we would have expected an age-dependent effect on the overall T cell response and not just the selective Th17 response that was in fact observed. Furthermore, we did not observe outliers amongst the 22, 35 and 57 year old control individuals in the ability of their T cells to produce IFN-γ or IL-17 following ex vivo stimulation and/or ex vivo differentiation.

Recent evidence suggests IL-17+ cells may be important very early in CF lung disease. Increased numbers of Th17 cells have been demonstrated in the submucosa of endobronchial biopsies of newly diagnosed CF infants and young children [12]. Furthermore, the numbers of total IL-17+ cells (including Th17, IL-17+ neutrophils, γδT and NKT cells) are increased in newly diagnosed CF subjects with bronchoalveolar lavage fluid neutrophilia in the absence of IL-8 [12]. We have identified a phenotype unique to naïve helper T cells from CF subjects, attributable to loss of CFTR expression, which predisposes them selectively towards Th17 differentiation while retaining a normal propensity to differentiate into other lineages such as Th1 and Treg. Our findings suggest that the observations of a Th17 response during the early onset of CF lung disease may be attributable to preferential differentiation of naïve T cells along the Th17 lineage. Moreover, the present study confirms a role of CFTR in modulating T cell biology by regulating differentiation along the Th17 lineage. However, the molecular pathways by which CFTR regulates Th17 differentiation remain to be elucidated.

## Conclusions

Taken together, our findings are to our knowledge the first to indicate an intrinsic phenotype of CF CD4+ T cells, attributable to loss of CFTR expression, which predisposes them towards selective Th17 differentiation while retaining a normal ability to undergo differentiation into Th1 and Treg lineages. These findings may account for the initiation of the Th17 response, amplified by the local cytokine milieu, that is associated with a clinical exacerbation of CF pulmonary pathology [11,13,26]. Therefore, direct modulation of T cells may hold potential as a novel therapeutic strategy in CF.

## Abbreviations

CF: Cystic fibrosis; CFTR: CF transmembrane conductance regulator; IL: Interleukin; Th0 cells: Naїve helper T lymphocytes.

## Competing interests

The authors declare that they have no competing interests.

## Authors’ contributions

RK designed and conducted the immunological analysis of the T cell responses in the peripheral blood of mice and human subjects, collected and interpreted data, and wrote most of the first draft of the paper. SG made substantial contributions to the acquisition of data. NBS drafted parts of the discussion. All authors were involved in revising the manuscript critically for important intellectual content, and read and approved the final manuscript.

## References

[B1] Riordan JR Rommens JM Kerem B Alon N Rozmahel R Grzelczak Z Zielenski J Lok S Plavsic N Chou JL Identification of the cystic fibrosis gene: cloning and characterization of complementary DNA Science 1989 14 1066 1073 10.1126/science.2475911 2475911

[B2] Cutting GR Kasch LM Rosenstein BJ Zielenski J Tsui LC Antonarakis SE Kazazian HH Jr A cluster of cystic fibrosis mutations in the first nucleotide-binding fold of the cystic fibrosis conductance regulator protein Nature 1990 14 366 369 10.1038/346366a0 1695717

[B3] Stutts MJ Canessa CM Olsen JC Hamrick M Cohn JA Rossier BC Boucher RC CFTR as a cAMP-dependent regulator of sodium channels Science 1995 14 847 850 10.1126/science.7543698 7543698

[B4] Dong YJ Chao AC Kouyama K Hsu YP Bocian RC Moss RB Gardner P Activation of CFTR chloride current by nitric oxide in human T lymphocytes EMBO J 1995 14 2700 2707 7540975 10.1002/j.1460-2075.1995.tb07270.x PMC398388

[B5] Kerem E Reisman J Corey M Canny GJ Levison H Prediction of mortality in patients with cystic fibrosis N Engl J Med 1992 14 1187 1191 10.1056/NEJM199204303261804 1285737

[B6] Chmiel JF Konstan MW Inflammation and anti-inflammatory therapies for cystic fibrosis Clin Chest Med 2007 14 331 346 10.1016/j.ccm.2007.02.002 17467552

[B7] Lyczak JB Cannon CL Pier GB Lung infections associated with cystic fibrosis Clin Microbiol Rev 2002 14 194 222 10.1128/CMR.15.2.194-222.2002 11932230 PMC118069

[B8] Singh PK Schaefer AL Parsek MR Moninger TO Welsh MJ Greenberg EP Quorum-sensing signals indicate that cystic fibrosis lungs are infected with bacterial biofilms Nature 2000 14 762 764 10.1038/35037627 11048725

[B9] Stockinger B Veldhoen M Martin B Th17 T cells: linking innate and adaptive immunity Semin Immunol 2007 14 353 361 10.1016/j.smim.2007.10.008 18023589

[B10] Dubin PJ Kolls JK IL-23 mediates inflammatory responses to mucoid Pseudomonas aeruginosa lung infection in mice Am J Physiol Lung Cell Mol Physiol 2007 14 L519 L528 17071720 10.1152/ajplung.00312.2006 PMC2841977

[B11] McAllister F Henry A Kreindler JL Dubin PJ Ulrich L Steele C Finder JD Pilewski JM Carreno BM Goldman SJ Role of IL-17A, IL-17F, and the IL-17 receptor in regulating growth-related oncogene-alpha and granulocyte colony-stimulating factor in bronchial epithelium: implications for airway inflammation in cystic fibrosis J Immunol 2005 14 404 412 15972674 10.4049/jimmunol.175.1.404 PMC2849297

[B12] Tan HL Regamey N Brown S Bush A Lloyd CM Davies JC The Th17 pathway in cystic fibrosis lung disease Am J Respir Crit Care Med 2011 14 252 258 10.1164/rccm.201102-0236OC 21474644 PMC3381840

[B13] Tiringer K Treis A Fucik P Gona M Gruber S Renner S Dehlink E Nachbaur E Horak F Jaksch P A Th17- and Th2-skewed cytokine profile in cystic fibrosis lungs represents a potential risk factor for Pseudomonas aeruginosa infection Am J Respir Crit Care Med 2013 14 621 629 10.1164/rccm.201206-1150OC 23306544

[B14] Iannitti RG Carvalho A Cunha C De LA Giovannini G Casagrande A Zelante T Vacca C Fallarino F Puccetti P Th17/Treg imbalance in murine cystic fibrosis is linked to indoleamine 2,3-dioxygenase deficiency but corrected by kynurenines Am J Respir Crit Care Med 2013 14 609 620 10.1164/rccm.201207-1346OC 23306541

[B15] Gibson LE Cooke RE A test for concentration of electrolytes in sweat in cystic fibrosis of the pancreas utilizing pilocarpine by ionophoresis Pediatr 1959 14 545 549 13633369

[B16] Constant S Pfeiffer C Woodard A Pasqualini T Bottomly K Extent of T cell receptor ligation can determine the functional differentiation of naive CD4+ T cells J Exp Med 1995 14 1591 1596 10.1084/jem.182.5.1591 7595230 PMC2192213

[B17] Segura E Touzot M Bohineust A Cappuccio A Chiocchia G Hosmalin A Dalod M Soumelis V Amigorena S Human inflammatory dendritic cells induce Th17 cell differentiation Immunity 2013 14 336 348 10.1016/j.immuni.2012.10.018 23352235

[B18] Jung T Schauer U Heusser C Neumann C Rieger C Detection of intracellular cytokines by flow cytometry J Immunol Methods 1993 14 197 207 10.1016/0022-1759(93)90158-4 8445253

[B19] Okamura H Tsutsi H Komatsu T Yutsudo M Hakura A Tanimoto T Torigoe K Okura T Nukada Y Hattori K Cloning of a new cytokine that induces IFN-gamma production by T cells Nature 1995 14 88 91 10.1038/378088a0 7477296

[B20] Hori S Nomura T Sakaguchi S Control of regulatory T cell development by the transcription factor Foxp3 Science 2003 14 1057 1061 10.1126/science.1079490 12522256

[B21] Ivanov II McKenzie BS Zhou L Tadokoro CE Lepelley A Lafaille JJ Cua DJ Littman DR The orphan nuclear receptor RORgammat directs the differentiation program of proinflammatory IL-17+ T helper cells Cell 2006 14 1121 1133 10.1016/j.cell.2006.07.035 16990136

[B22] Gregory RJ Cheng SH Rich DP Marshall J Paul S Hehir K Ostedgaard L Klinger KW Welsh MJ Smith AE Expression and characterization of the cystic fibrosis transmembrane conductance regulator Nature 1990 14 382 386 10.1038/347382a0 1699127

[B23] Rich DP Anderson MP Gregory RJ Cheng SH Paul S Jefferson DM McCann JD Klinger KW Smith AE Welsh MJ Expression of cystic fibrosis transmembrane conductance regulator corrects defective chloride channel regulation in cystic fibrosis airway epithelial cells Nature 1990 14 358 363 10.1038/347358a0 1699126

[B24] Chambers CA The expanding world of co-stimulation: the two-signal model revisited Trends Immunol 2001 14 217 223 10.1016/S1471-4906(01)01868-3 11274928

[B25] Tsoukas CD Landgraf B Bentin J Valentine M Lotz M Vaughan JH Carson DA Activation of resting T lymphocytes by anti-CD3 (T3) antibodies in the absence of monocytes J Immunol 1985 14 1719 1723 3926881

[B26] Chan YR Chen K Duncan SR Lathrop KL Latoche JD Logar AJ Pociask DA Wahlberg BJ Ray P Ray A Patients with cystic fibrosis have inducible IL-17 + IL-22+ memory cells in lung draining lymph nodes J Allergy Clin Immunol 2013 14 1117 1129 10.1016/j.jaci.2012.05.036 22795370 PMC3488163

[B27] Chen W Jin W Hardegen N Lei KJ Li L Marinos N McGrady G Wahl SM Conversion of peripheral CD4 + CD25- naive T cells to CD4 + CD25+ regulatory T cells by TGF-beta induction of transcription factor Foxp3 J Exp Med 2003 14 1875 1886 10.1084/jem.20030152 14676299 PMC2194145

[B28] Ziegler SF FOXP3: of mice and men Annu Rev Immunol 2006 14 209 226 10.1146/annurev.immunol.24.021605.090547 16551248

[B29] Mackay CR Dual personality of memory T cells Nature 1999 14 659 660 10.1038/44309 10537102

[B30] Cohen-Cymberknoh M Kerem E Ferkol T Elizur A Airway inflammation in cystic fibrosis: molecular mechanisms and clinical implications Thorax 2013 10.1136/thoraxjnl-2013-203204 23704228

[B31] Corvol H Fitting C Chadelat K Jacquot J Tabary O Boule M Cavaillon JM Clement A Distinct cytokine production by lung and blood neutrophils from children with cystic fibrosis Am J Physiol Lung Cell Mol Physiol 2003 14 L997 1003 12547728 10.1152/ajplung.00156.2002

[B32] Moss RB Hsu YP Olds L Cytokine dysregulation in activated cystic fibrosis (CF) peripheral lymphocytes Clin Exp Immunol 2000 14 518 525 10844532 10.1046/j.1365-2249.2000.01232.x PMC1905557

[B33] Li G Yu M Lee WW Tsang M Krishnan E Weyand CM Goronzy JJ Decline in miR-181a expression with age impairs T cell receptor sensitivity by increasing DUSP6 activity Nat Med 2012 14 1518 1524 10.1038/nm.2963 23023500 PMC3466346

